# Influence of Training Load on the Risk of Injuries in Preprofessional Contemporary Dancers: A Scoping Review

**DOI:** 10.1177/23259671251369004

**Published:** 2025-11-01

**Authors:** Chenée Armando, Sai S. Kurapati, Morgan A. Voulo, Robert A. Gallo

**Affiliations:** † Department of Orthopaedics and Rehabilitation, Penn State Milton S. Hershey Medical Center, Hershey, Pennsylvania, USA; Investigation performed at Pennsylvania State College of Medicine, Hershey, Pennsylvania, USA

**Keywords:** contemporary dance, dance, female athlete, medical aspects of sport, pediatric sports medicine

## Abstract

**Background:**

Contemporary dance consists of blending many dance styles and exploring movements that test the physical boundaries of the human form. A preprofessional contemporary dancer can have an intense training load as a performance athlete. Despite intense training, the detailed factors influencing injury are not completely understood.

**Purpose:**

To investigate how training intensity, type, and duration can affect the incidence and severity of injury in preprofessional contemporary dancers.

**Study Design:**

Scoping review; Level of evidence, 4.

**Methods:**

Following the PRISMA (Preferred Reporting Items for Systematic Reviews and Meta-Analyses) guidelines, 6 databases were searched in July 2022, yielding 226 relevant records. The search query used broad terms to capture as many studies as possible: danc* AND contemporary dance AND injury. After initial screening, 111 studies underwent abstract review, resulting in 54 being selected for full-text review. Finally, 9 studies were included in this review, and a qualitative synthesis was conducted.

**Results:**

Three themes emerged from the studies in this review. First, there was a lack of standardization in the definitions of injury across the studies. These variations affected the comprehensive assessment of the overall effect of high-level training on contemporary dancers. Second, a high incidence of overuse injuries was reported. Overuse injuries ranged from 20% to 80% of the total injuries that were reported. Third, a sudden spike in training hours during the final weeks leading up to performances resulted in a higher incidence of injuries, which yielded a statistically significant nonlinear correlation across multiple studies. This was confirmed by applying a cubic spline model on the aggregated data, which yielded results of 99.7% of the variance in injury incidence based on training load that were similarly significant (*P* = .037).

**Conclusion:**

Our study showed that despite the lack of standardization in defining "injury" among preprofessional dancers, an inclusive definition encompassing “all complaints” proves most accurate for reporting injury incidence among contemporary dancers, who frequently suffer from overuse injuries. Erratic training schedules—particularly rapid increases in weekly training hours—significantly contribute to heightened injury rates. These data could contribute to the development of preventive guidelines for athletes engaged in high-intensity training.

Contemporary dance consists of a combination of many dance styles and explores movements that test the physical boundaries of the human form. While ballet is commonly considered the foundation of most dance styles, contemporary dance is not limited to the strict rules of ballet technique. This dance form is shaped by the modern dance innovations of Isadora Duncan, Martha Graham, and Merce Cunningham.^13,43^ Duncan emphasized natural, expressive movement, Graham introduced contraction and release for emotional storytelling, and Cunningham prioritized pure movement and chance choreography, all of which influenced its evolution.^13,43^ It blends styles from around the world and experiments with rapid explosive movement, changes in body alignment, and one's own breathing to express stories and emotions. Although the contemporary style allows for freedom of expression, the training involved at high levels is demanding and follows a rigorous schedule. It is an art form that is physically taxing, and like all athletic pursuits, puts the practitioners at risk of injury.^13,43^

The prevalence of injury in ballet dancers is widely documented.^12,26,30,38^ While ballet and contemporary dance share similarities, their distinct styles contribute to varying injury risks. Ballet's use of pointe shoes necessitates extreme plantarflexion, coupled with hip hypermobility, making ballet dancers susceptible to specific injuries like ankle tendinopathy, snapping hip syndrome, and femoroacetabular impingement. For young dancers with underdeveloped strength, the risk for ankle ligament injuries is particularly pronounced.^35^ As dancers progress, the exposure to extreme ranges increases, elevating the potential for chronic tendinopathies. In contrast, contemporary dancers often perform barefoot or with minimal sole support and variable landing from dynamic jumps, which changes the type of specific foot-related injuries. Moreover, classical ballet's strict body part placement serves both aesthetic and protective purposes. At the same time, contemporary dance intentionally breaks these rules, incorporating movements that extend beyond traditional ballet lines—including increased hip and spine range of motion.^1,15,40^ Furthermore, ballet technique emphasizes elongation and soft landings to create the illusion of floating. In contrast, contemporary technique, influenced by modern dance, is more grounded and involves increased contact with the floor, resulting in probable stresses on the joints. However, limited research is available regarding injuries among contemporary dancers, despite their increasing popularity, due in part to the development of social media platforms like YouTube and the TV show, “So You Think You Can Dance.”^3,29^ A preprofessional contemporary dancer can have up to double the training load of a professional soccer player.^19^ As in any elite athletic training routine, an increased training load correlates with an increased risk of musculoskeletal injury.^20^ However, the detailed factors that influence injury incidence, type, or severity among contemporary dancers are not completely understood.

Greater knowledge of injury risk can potentially help mitigate and decrease its incidence. The best way to collect these data is to use an accurate and uniform reporting system across research, such as those created by other athletic organizations, such as the National Collegiate Athletic Association.^27^ While the International Association for Dance Medicine & Science provides a standardized method for reporting injuries in dancers, not all studies have adopted these definitions.^8,9,11,16,21,25,30,31,41^ This systematic review analyzes studies that have investigated injuries in contemporary dance and differences in the reporting systems. This systematic review aimed to investigate how training intensity, type, and duration can affect the type, risk, and severity of injury in preprofessional contemporary dancers. The hypothesis was that pattern variations in training would change the likelihood and severity of injury in this population.

## Methods

### Search Strategy

**Figure 1.**
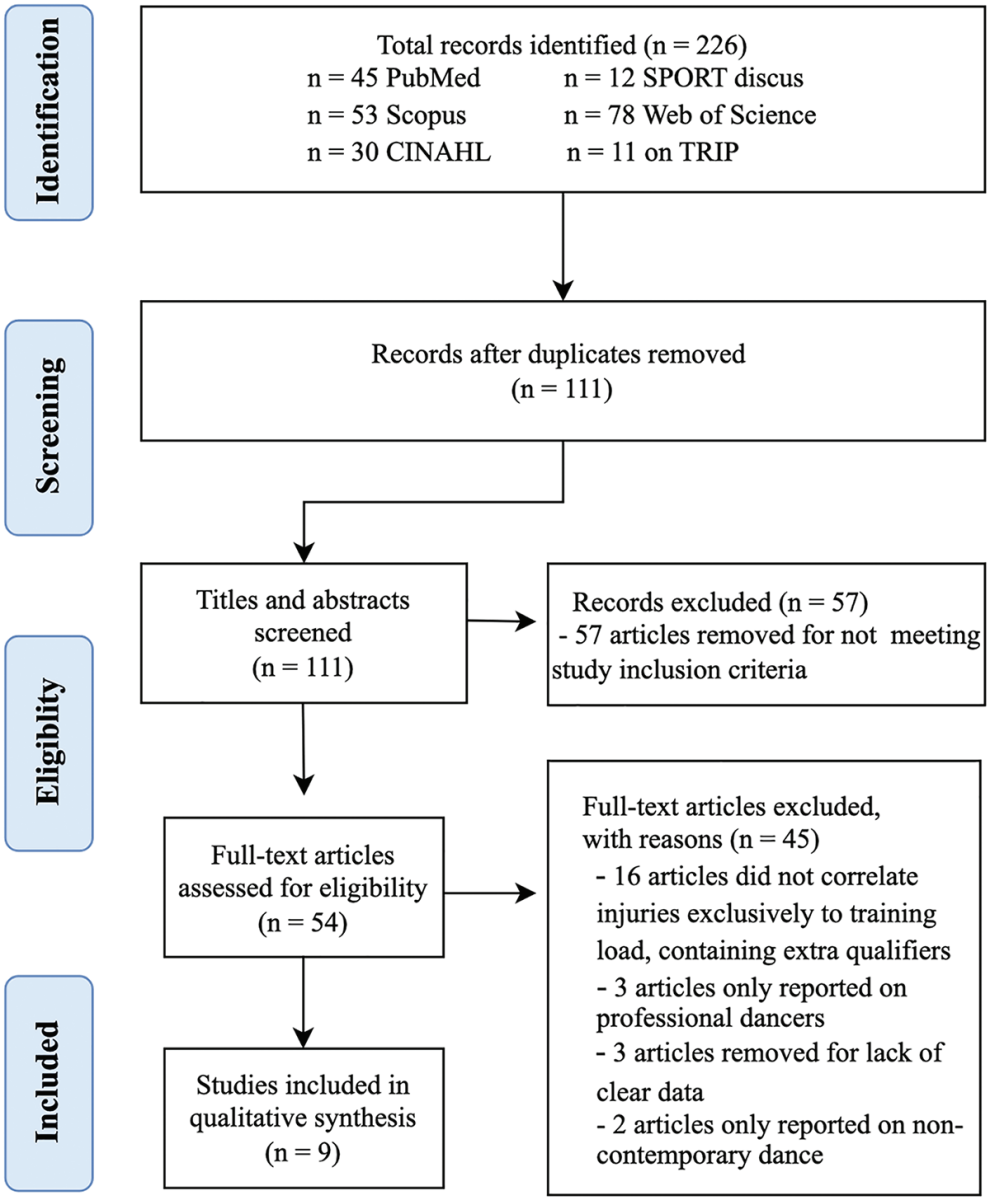
PRISMA flow diagram. PRISMA, Preferred Reporting Items for Systematic Reviews and Meta-Analyses.

A systematic literature search was conducted according to the PRISMA (Preferred Reporting Items for Systematic Reviews and Meta-Analyses) guidelines (Figure 1). To extract relevant literature, we searched PubMed, Scopus, CINAHL, SPORTdiscus, Web of Science, and TRIP databases for articles published between January 2005 and the date of search, July 2022. These databases are recognized as significant sources of high-quality research on sports and exercise science. The following search query was carefully developed to locate articles specific to this study on the influence of training load on the risk of injuries in contemporary dancers: danc* AND contemporary dance AND injury. Combinations of other supplemental mesh terms such as “Dancing/adverse effects”[Mesh] OR “Dancing/education”[Mesh] OR “Dancing/injuries”[Mesh] OR “Dancing/physiology”[Mesh] were also tested. However, this search query captured optimal data specificity while maintaining the broad scope required for a comprehensive analysis. The initial review of search results was conducted by a physician (C.A.) who is a graduate of the American Ballet Theatre Jacqueline Kennedy Onassis School of Ballet, with professional experience. Discrepancies were resolved through discussion among all authors, and duplicates were removed.

#### Criteria for Study Inclusion and Exclusion

The inclusion criteria were as follows: (1) studies reporting on a range of injury characteristics (such as location, severity, traumatic event, or chronic); (2) research conducted after 2005; (3) preprofessional programs; (4) research that documented training time, load, or dance exposure; and (5) articles published in the English language. We excluded studies from this review that did not provide data on (1) training time or (2) injury incidence correlated with factors apart from training loads, such as lower body strength, mobility, or mental health. While recognizing that these factors have an important role in how the body responds to stress, including them poses the risk of excessive confounding factors. These carefully designed eligibility criteria were enforced to ensure that only studies appropriate for the objectives of this systematic literature review were included in our analysis.

#### Study Selection and Data Extraction

Our advanced search query applied across the 6 databases resulted in 226 relevant records overall (45 on PubMed, 53 on Scopus, 30 on CINAHL, 12 on SPORTdiscus, 78 on Web of Science, and 11 on TRIP). An initial screening was then conducted to eliminate duplicate articles and identify articles that superficially fit our inclusion criteria. This yielded 111 studies in total, which underwent abstract review. The remaining 54 studies were selected for full-text review. Three independent reviewers (C.A., S.S.K., M.A.V.) with training in systematic review methodology and/or professional dance experience conducted the final in-depth qualitative synthesis of the manuscripts. All final decisions were reviewed and approved by the primary investigator (R.A.G.), who is a fellowship-trained, board-certified orthopaedic sports medicine surgeon. This resulted in 9 studies that were included for analysis in this review, which examined injury incidence, type, training load, and severity of injury, followed by a summary of the available epidemiologic data.

## Results

A total of 9 studies were included in this analysis.^8,9,11,16,21,25,30,31,41^

### Study Characteristics

A summary of the extracted study characteristics (author, year published, title, study design, sample size, and study duration), training characteristics (training type, duration, and intensity), and injury characteristics (including the number of injuries, location, type, risk, and severity) are included in Tables 1 and 2. Three main themes emerged among the 9 studies included in this review (Table 3).

**Table 1:** Study Characteristics*^*a*^*

Author/Year	Method	Injury Definition	Sample Size	Follow-up Duration
Cahalan et al^9^ (2018)	Prospective cohort	Pain or injury that impacted the ability to dance	29	42-52 wk
van Winden et al^41^ (2019)	Prospective cohort	Non-time loss injuries and time loss injuries	130	1 yr
Mailuhu et al^30^ (2021)	Prospective cohort	Non-time loss injuries and time loss injuries	91	1 yr
Cahalan et al^8^ (2019)	Prospective cohort	Any pain or injury	30	1 yr
Kenny et al^21^ (2018)	Prospective cohort Study	(1) Time loss; (2) medical attention; (3) any complaint	60	1 yr
Fuller et al^16^ (2020)	Retrospective cohort	injury requiring medical attention	17	3 yrs
Lai et al^25^ (2022)	Cross-sectional study	(1) An anatomic tissue-level impairment that occurred as a result of participation in dance; (2) An anatomic tissue-level impairment that resulted in suboptimal performance	10	10 mo
Campoy et al^11^ (2011)	Cross-sectional study, retrospective	Any pain or injury that impacted the ability to dance	115	1 yr
Nunes et al^31^ (2022)	CART analysis	Full time loss (an inability to participate fully in training or competition)	13	1 yr

a CART, classification and regression tree.

**Table 2:** Training and Injury Characteristics*^*a*^*

Training Characteristics			Injury Characteristics			
Author/year	Duration of training	No. of injuries	Location of injury	Injury type*^*b*^*	Risk of injury*^*c*^*	Severity of injury*^*d*^*
Cahalan et al^9^ (2018)	12.17 hrs/wk	155	Ankle/foot, 12.9; knee, 17.4; low back, 9.7; other, 60	Overuse, 20.1;	8.4 injuries per 1000 hrs	2.4 time-loss injuries per 1000 hrs
van Winden et al^41^ (2019)	Total 132,906 dance exposure hrs per year	321	Ankle/foot, 30; knee, 15; low back, 17; other, 38	Not specified	1.9 injuries per 1000 hrs	58.1 time-loss injuries
Mailuhu et al^30^ (2021)	Total 96,047.25 dance exposure hours per year; 1055.5 ± 276.2 hours per year per student	33	This study only assessed ankle injuries	Not specified	0.24 injuries per 1000 hrs	15.4 time-loss injuries
Cahalan et al^8^ (2019)	21 hrs per wk	45	Ankle/foot, 25; knee, 20.5; low back, 9.1; other, 45.4	New, 15 (33.3); recurring, 29 (64.5)	Not specified	46.7 time loss injuries, 1-7 days (mild); 24.4 time loss injuries, 8-21 days (moderate); 26.7 time loss injuries, 21+ days (severe); 2.2 not stated
Kenny et al^21^ (2018)	Total 26363.45 hours per 31 wks	Injury prevalence proportion (contemporary only): Time loss: 26.7; medical attention: 50; all complaints: 78.3	Not specified	Not specified	1. 0.91 injuries per 1000 hrs; 2. 0.87 injuries per 1000 hrs; 3. 4.89 injuries per 1000 hrs	1. 125 days lost; 2. 153 days lost; 3. 139 days lost
Fuller et al^16^ (2020)	30 hrs per wk for 28 wks per yr	119	Ankle, 17.65; knee, 16.81; other, 65.54;	Acute, 6	2.71 injuries per 1000 hrs	0.07 time loss injuries per 1000 hrs
Lai et al^25^ (2022)	Total 18,290 dance exposure hrs per year; mean of 11 hrs per week		This study only assessed ankle and foot injuries	Recurring, 80	0.38 injuries per 1000 hrs	Not specified
Campoy et al^11^ (2011)	9.38 ± 8.34 hr/wk (absence of injury), 15.84 ± 12.49 hr/wk (presence of injury)	89	Ankle/foot, 21.43%; knee, 21.43%; other, 57.14%	Acute, 62.98; overuse, 37.01;	1.33 injuries per participant; 1.73 injuries per injured participant	Not specified
Nunes et al^31^ (2022)	13.5 ± 12.9 hr/wk	20	Not specified	Not specified	52.4 incidence of injury with a workload > 11.5 hrs/wk; 20 incidence of injury with a workload of ≤11.5 hrs/wk.	Not specified

a Data are presented as n, n (%), or %.

b Overuse/recurring versus traumatic/acute.

c Risk of injury (incidence/1000 hrs).

d Severity (reduction in training volume/time) of injury.

**Table 3:** Common Themes in Analyzed Studies*^*a*^*

	Definition of Injury	Overuse Injury Incidence	Variable Training Schedule
Cahalan et al^9^ (2018)	✓	-	✓
van Winden et al^41^ (2019)	✓	✓	-
Mailuhu et al^30^ (2021)	✓	✓	-
Cahalan et al^8^ (2019)	-	✓	-
Kenny et al^21^ (2018)	✓	✓	-
Fuller et al^16^ (2020)	✓	✓	✓
Lai et al^25^ (2022)	✓	✓	-
Campoy et al^11^ (2011)	✓	✓	✓
Nunes et al^31^ (2022)	✓	-	✓

a ✓: Topic discussed within the results of the text. -: Topic not discussed within the results of the text.ta.

### Definition of Injury

The first theme focuses on the lack of standardization in the definitions of injury across the studies. These variations affected the comprehensive assessment of the overall effect of high-level contemporary dance. For example, Fuller et al^16^ defined injury in the context of needing medical attention. Participants in this study had a medical clinic where they could seek care for their injuries; it was available once per week. They reported an injury incidence of 2.71 per 1000 hours, the lowest reported in this review.

Kenny et al^21^ compared definitions of injury to determine which would accurately capture the incidence of injury among ballet and contemporary dancers. They hypothesized that the narrow definition of time lost to injury and injuries requiring medical attention would underrepresent the effect of pain and overuse injuries on the athletes. Therefore, Kenny et al outlined 3 separate definitions of injury—including “time loss,”“medical attention,” and “all complaints.” The results indicated that the use of the “all complaints” definition, particularly for overuse injuries, resulted in a more comprehensive inclusion of cases. The “all complaints” category had an injury incidence of 4.9 per 1000 hours, while the “time loss” category had an injury incidence of 0.91 per 1000 hours, and the “medical attention” category had 0.87 per 1000 hours. To confirm the validity of the “all complaints” definition, Kenny et al included days lost from dance due to injury; “all complaints” resulted in 139 days lost from injury, “time loss” resulted in 125 days lost from injury, and the “medical attention” category resulted in 153 days lost from injury.

Six of the studies that were reviewed referenced the work of Kenny et al^21^ to create their definition of injury, and 1 of the studies referenced an earlier paper by the authors.^8,9,16,30,31,41^ Cahalan et al^9^ defined injury as “any pain or injury that impacted upon their ability to dance.” This study reported an injury incidence of 8.4 injuries per 1000 hours. This was the highest injury incidence reported among all the studies included in this review. Van Winden et al^41^ separated their categories of injury as “Non-time loss injuries” and “time loss injuries.”^41^ The researchers administered the study using the Oslo Sports Trauma Research Centre Overuse Injury Questionnaire. They reported only the most severe injuries that occurred each month, and they reported no more than 1 injury per month for each participant. This resulted in a reduced incidence value of 1.9 injuries per 1000 hours. The researchers also included an “all-injury” value that increased the incidence of 3.04 injuries per 1000 hours. Campoy et al^11^ defined injury as any pain or hindrance that impacted the dancers’ normal training routine. This study assessed the mechanism of injury and did not account for the number of training hours.

Two of the studies assessed only ankle and foot injuries.^25,30^ They reported 0.24 ankle injuries per 1000 hours of dance exposure and 0.38 ankle and foot injuries per 1000 hours of dance, respectively. The injury was defined as tissue impairment leading to compromised performance for the dancers.^25^

### Incidence of Overuse Injury

The second theme is centered around the high incidence of overuse injuries. The chosen definition of each study was a determining factor in accurately capturing overuse injuries. Seven studies endorsed that definitions such as “time loss” to injury and “medical attention” to injury fail to document many overuse injuries.^8,11,16,21,25,30,41^ Cahalan et al^9^ reported that 20.1% of injuries were due to overuse, and 70.4% of injuries were non-time-loss injuries. Cahalan et al^25^ reported that 64.5% of injuries were recurring injuries ^8^; Lai et al^25^ reported that 80% of injuries were recurring events. Campoy et al^11^ reported that 37.01% of injuries were due to excessive use. Fuller et al^16^ categorized mechanisms of injuries, noting that 6% of the injuries were traumatic.

### Impact of Training Schedule

**Figure 2.**
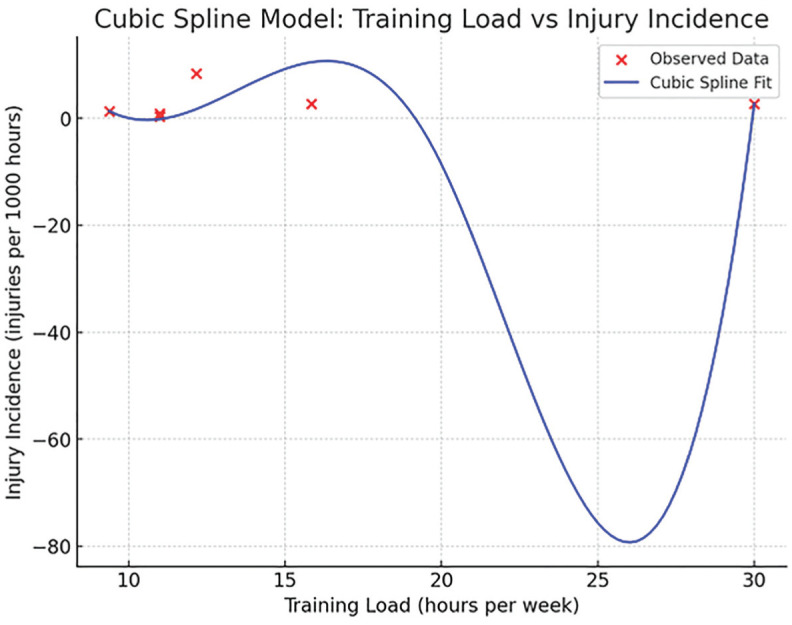
Cubic spline regression: injury incidence vs training load.

The third theme correlated the dancers’ training schedule with injury incidence. Four studies described an abrupt increase in training intensity and duration due to an upcoming performance, which preceded an increase in injury incidence.^9,11,16,31^ Cahalan et al^9^ noted that an increased injury incidence was preceded by a spike in training hours the week prior by a mean of 3.7 hours (totaling a mean of 18.9 [±7.5] hours of training load). A sporadic training schedule that included sharp increases in training hours on certain weeks, without a gradation, was also reported.^16^ The sudden spikes in training hours were predictable, as they consistently occurred in the weeks leading up to performances. This pattern showed a statistically significant nonlinear correlation between training load and injury risk in the study's results,^11,31^ as well as a statistically significant nonlinear correlation in our calculations using the cubic spline model (Figure 2). The data set of training load (hrs/week) {12.17, 30, 11, 9.38, 15.84, 11} versus injury incidence (per 1000 hrs) {8.4, 2.71, 0.38, 1.33, 2.71, 0.91}^9,11,16,21,25^ showed 99.7% of the variance in injury incidence based on training load (*P* = .037). Higher training loads (>11.5 hrs/wk) were significantly correlated with severe pain intensity and injury incidence.^31^ Several studies suggested the incorporation of a gradual progression program into the curriculum, although none of the reviewed studies specified a particular protocol.^11,16,31^

## Discussion

The most revealing finding consistent among most of the 9 papers in this review is regarding the irregular training schedules, which predisposed dancers to increased injury incidence.^8,9,11,16,21,25,30,31,41^ This practice was prevalent among most of the contemporary dance programs included in this review. Before the onset of the increased injury incidence, there were sudden changes in the workload. This spike was likely due to either preparation for an upcoming performance or returning from a scheduled break without gradually building back up to a full schedule.^9,11,16,31^ A sudden increase in training likely did not provide enough time for the dancers to make the necessary physiological compensations to handle the demands of high dynamic load and repetitive stress. Some studies recommended that a steady gradation program be implemented in the curriculum.^11,16,31^ We believe that preprofessional dancers can protect themselves by implementing calibrated supplemental training outside of technique classes and rehearsals. For example, incorporating strength, mobility, endurance, and plyometric training outside the normal rehearsal schedule can help mitigate the risk of injuries.^32,33^ Strength training modalities should replicate the range of motion, angle, and movement patterns needed for contemporary dance. This improves the peripheral muscle fiber modifications necessary to execute specific skills.^33^ Supplemental strength training should be introduced in a graduated manner and designed to complement the dance training from the school curriculum.^23,28^ The goal of dance programs should be to create a schedule that adapts when rehearsal and performance demands increase, thereby balancing the stress on the dancers’ bodies. For example, ramping up strength training in the weeks before the more intense preperformance specific training, and then decreasing the supplemental strength training intensity and focusing on therapeutic exercises during the preperformance period.^32^ Additional care should be taken to ensure adequate nutrition and sleep, particularly during these intense training periods.^32^ In theory, it may be useful to consider something akin to a “pitch count” such as a “step count.” However, this is complicated by the sheer number of different steps and the various stresses they impose on the body. Therefore, a complementary supplemental training program that counterbalances the intensity of the rehearsal periods may be a more practical approach to injury prevention.

Musculoskeletal injuries arise from intrinsic factors, extrinsic factors, or both.^28^ An improved understanding of the spectrum of injuries that dancers experience can help inform dance organizations, educators, and advising medical professionals on how to mitigate the various risk factors, thereby improving dancers' career longevity. An important first step in understanding these risk factors is standardizing a definition of injury that accurately captures the dancers’ experience and risk of exposure within contemporary dance. Many of the studies included in this review referenced the work of Kenny et al^21^ when discussing their approach to defining the term “injury” in their respective studies; the 3 definitions compared were “time loss to injury,”“medical attention to injury,” and “all complaints.” Kenny et al concluded that the most inclusive “all complaints” definition was the most preferred to use, especially when attempting to document overuse and chronic injuries. Fuller et al^16^ used the “medical attention” definition in their reporting, conceded that the lack of available medical professionals may have accounted for underreported injuries and underestimated injury incidence.

The severity of the injury is typically measured by days lost per 1000 hours of dance exposure.^19,41^ While this reporting method has become the most widely utilized in recent dance literature, it still falls short of capturing the overall consequence of injury within contemporary dance.^4,19,41^ Many of the studies in this review discussed the prominence of overuse injuries. The weighted mean of the total reported overuse injuries in this review was 57%. The reported^12^ prevalence among some other sports ranges from 16% to 39%. Despite the high prevalence, it was also concluded that overuse injuries remain underreported.^25^ Functional measurements—such as stability, pain levels, range of motion, and strength—may provide more accurate insights into the long-term consequences of the injury, as opposed to simply documenting the number of days lost. ^2^ This is especially important as dancers move from the collegiate world to the professional arena, where days lost to injury are less of an option, and a high percentage of dancers continue to perform through pain and injury.

While the inclusion of professional dancers may have provided additional useful data, the increased performance demands on professional dancers would likely skew the analysis and limit the application of data. While preprofessional dancers may face intrinsic pressure to push their limits, not take time off, and train through pain, professional dancers face extrinsic pressures that push their limits. Many professional companies, particularly smaller ones with limited funding, do not pay dancers for their time off stage and do not offer medical insurance.^34,42^ Therefore, dancers’ compensation is often dependent on avoiding days off from dancing. It is therefore difficult to have an accurate depiction of injury if the measurement of severity is days lost instead of a measure of functionality. A stark example of this is seen in a study that followed professional dancers^5^; in this study, every professional contemporary dancer reported having a current injury, but none reported time lost to injury. Therefore, it is important to have a comprehensive understanding of acute, chronic, and overuse injuries, which are not adequately measured by time lost to injury. Accurately documenting the functional abilities of contemporary dancers at different stages in their careers, as well as identifying reasons for early retirement from dance, can inform strategies to mitigate risks and improve musculoskeletal health.

A more comprehensive understanding of injury risk in dancers may be achieved by analyzing the relationship between training type, intensity, and weekly injury incidence. Although training averages and overall incidences were reported,^16,19,41^ a detailed delineation of training schedules—including specific breakdowns of technique classes, rehearsals, and performances—remains limited.^5,18,32^ This distinction is critical, as each type of training imposes distinct biomechanical and physiological demands on the body.^4,33^ For example, a technique class includes dance combinations that are created to train the body bilaterally in an equal fashion.^32^ However, choreography for performance tends to favor the dominant lateral side of the group, which, as observed in previous studies, is frequently the right side.^32^

Rehearsals and back-to-back performances can lead to asymmetric loading, contributing to muscular imbalances, characterized by unilateral strength gains on the dominant side and increased mobility on the contralateral side, potentially predisposing dancers to overuse injuries.^5,6,7,25^ One example is the grand battement à la seconde; this step is classically seen in ballet, but variations are also executed regularly in contemporary, modern, and jazz.^6,7,9^ In this step, the working leg rapidly accelerates from a standing position into abduction beyond 135°, while maintaining knee extension.^6,7,24,36^ The iliopsoas and rectus femoris muscles of the working leg contract, while the adductors are stretched.^24,36^ At the movement's peak, the abductors stabilize the hip without reaching maximal contraction. Meanwhile, the standing leg fully contracts the hip abductors and the quadratus lumborum to stabilize the spine.^24,36^ Simultaneously, the standing leg eccentrically contracts the adductors, iliopsoas, and rectus femoris to their maximum length.^24,28,36^

During a technique class, the stress of this motion is offset because it is executed bilaterally a predetermined number of times.^7,8,21^ On the other hand, a rehearsal will often train one side of the body for multiple hours at a time, resulting in the risk of creating imbalances and ultimately overuse injuries.^5,16,25^ This assessment is still simplified, as it does not include variability in technique due to individual variation and proficiency level, all of which influence injury susceptibility.^8,9,21^ The textbook technique is very challenging for practitioners to accurately execute. These reasons highlight the importance of future research to include detailed documentation of training modalities and durations, alongside injury incidence, to appreciate the complete effect of contemporary dance on the human body.

Biomechanical imbalances due to favoring 1 unilateral side increase the risk of overuse and chronic injuries.^22,39^ It is useful for dance practitioners and dance educators to be aware of the spectrum of injury incidence and injury details, such as length of time to recovery, decreased functionality, and change in the quality of performance due to injury.^2^ Another useful marker in understanding the long-term effect of chronic injuries on dancers is to assess the reasons behind retirement. Unanswered questions to date include the incidence of retirement from dance due to injury, long-term pain, or procedures required years after performing. Understanding more about overuse and chronic injuries can help guide changes in vocational and professional training that thereby increase the longevity of an individual dancing at a high level. Research has yielded valuable insights into various aspects of overuse injuries among professional dancers. For instance, studies have explored dancers' perceptions of pain, the transition to retirement, and the influence of external factors. One study highlighted the effect of external pressure on injury rates.^17^ Moreover, reports have shed light on the cultural pressures within the dance world, where notions of pushing through pain and injury are intertwined with preserving one's reputation and avoiding being labeled as a “lazy dancer.”^4^ Additional research on professional dancers has shown that living with chronic pain became an overwhelming burden for many dancers.^5,16,37^ Nevertheless, the existing literature provides limited evidence on the long-lasting effects of injuries that continue to cause pain and require medical interventions years after dancers retire from their careers. Conducting further research in this area would contribute to uncovering the short- and long-term consequences of contemporary dance on dancers' well-being.

Ballet and contemporary dance exhibit distinct injury patterns. Ballet, with its specific footwear and adherence to certain techniques, is prone to ankle and hip injuries.^14,35,38^ Contemporary dance, influenced by ballet but with distinct footwear and elements like floorwork and leaps involving various take-off and landing angles, can elevate stress on joints, particularly the knee.^11^ Contemporary dance also differs in its floorwork, which increases contact between the knees, shins, and the ground. This contributes to a higher incidence of injuries,^8,9,11,16,41^ with knee issues accounting for a mean of 18%, alongside foot and ankle injuries at 19%. The injury distribution contrasts with ballet, which tends to have more ankle and hip injuries. However, while contemporary dance exhibits a comparable ratio of foot and ankle injuries to ballet, ballet dancers experience more Achilles, forefoot, and midfoot pathology. In contrast, contemporary dancers face a higher incidence of hindfoot and ankle issues.^25^ Notably, dancers often report lower back injuries as a common complaint, further differentiating their injury profile from that of ballet dancers.^8,9,41^

Future research should consist of a more robust assessment of training load, intensities, and dance schedules compared with injury incidence and type. Furthermore, the initial research question inquired about the effects of recovery time. However, no studies were found that included this information in their research. Recovery time would be a pertinent variable to assess, as there is research investigating recovery time and peak performance in other sports.^10^ Moreover, detailed information on retired professional contemporary dancers—including reasons for retirement, lingering chronic pain, and surgical procedures—is in all areas that require more investigation in future research.

### Limitations

This review has several limitations. First, the confines of the data reported varied between each study included in this review. For example, only 5 of the studies reported their injury incidence as injuries per 1000 hours. In addition, 6 of the articles did not report the injury in a uniform category, such as acute or overuse. These variations between the studies prohibited the inclusion of quantitative analyses within this review. Therefore, this review faces the inherent limitations of qualitative analysis. Although we utilized the reported consistent units of measurement to calculate weighted averages, these are meant to assist in drawing trends rather than reviewing statistical data. Second, the data included in the studies did not always provide detailed training schedules. While many studies mentioned the weekly hours and cumulative semester hours and listed the dance exposure modalities, they did not specify the number of hours dedicated to each training type per week. Gaining a comprehensive understanding of training schedules and intensities every week, along with detailed information on injury incidence, would offer a clearer perspective on which training modalities may have a protective effect against injury and which ones could increase the risk. Third, the small sample size of studies meeting the inclusion criteria may pose challenges to the generalizability of our conclusions. Fourth, this review did not include the psychosocial factors influencing injury reporting, especially in younger populations, where social and psychological barriers may affect disclosure. Finally, because many studies in this review focus on collegiate dancers who likely train in various styles, confounding variables may influence their injury patterns.

## Conclusion

Our studies show that despite the lack of standardization in defining “injury” among preprofessional dancers, an inclusive definition encompassing “all complaints” proves most accurate for reporting injury incidence among contemporary dancers, who frequently suffer from overuse injuries. Erratic training schedules—particularly rapid increases in weekly training hours—significantly contribute to heightened injury rates. These data could contribute to the development of preventive guidelines for athletes engaged in high-intensity training.
